# Kinetic Analysis of Glycerol Esterification Using Tin Exchanged Tungstophosphoric Acid on K-10

**DOI:** 10.1021/acs.iecr.2c01930

**Published:** 2022-10-26

**Authors:** John Keogh, Callum Jeffrey, Manishkumar S. Tiwari, Haresh Manyar

**Affiliations:** † School of Chemistry and Chemical Engineering, Queen’s University Belfast, David-Keir Building, Stranmillis Road, BelfastBT9 5AG, U.K.; ‡ Department of Chemical Engineering, Mukesh Patel School of Technology Management and Engineering, SVKM’s NMIMS University, Mumbai, 400056Maharashtra, India

## Abstract

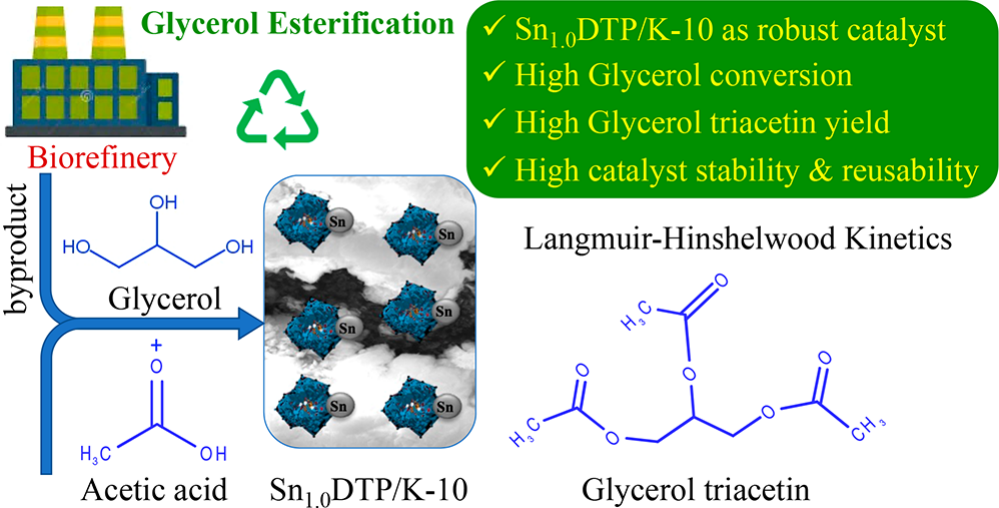

Glycerol acetins (mono-, di-, and tri) are produced via esterification with acetic acid. The acetins are commercially important industrial chemicals including their application as fuel additives, thus significant to environmental sustainability and economic viability of the biorefinery industry. Glycerol esterification with acetic acid was studied using partial tin exchanged tungstophosphoric acid supported on montmorillonite K-10 as catalysts. Partially exchanging the H^+^ ion of DTP with Sn (*x* = 1) increased the acidity of the catalyst and showed an increase in the catalytic activity as compared to the DTP/K-10 catalyst. A series of tin exchanged tungstophosphoric acid (20% w/w) supported on montmorillonite K-10 clay (Sn_*x*_-DTP/K-10, where *x* = 0.5–1.5) were synthesized and thoroughly characterized by using BET, XRD, FT-IR, UV–vis, and titration techniques. Among various catalysts, Sn_1_-DTP/K-10 was found to be the most active catalyst for glycerol esterification. Effects of different reaction parameters were studied and optimized to get high yields of glycerol triacetin. A suitable kinetic model of the reaction was fitted, and the Langmuir–Hinshelwood (L-H) dual-site model was able to describe the experimental data with high agreement between the experimental and calculated results. The prepared catalyst could be recycled at least four times without significant loss of activity. The overall process is green and environment friendly.

## 1 Introduction

With energy contributing 60% of total greenhouse gas emissions (UN), there has been a global drive towards more sustainable and renewable energy sources, with an ambition to reach net zero by 2050. As such, affordable and clean energy is number 7 on the United Nation’s sustainable development goals. In the transportation sector, this drive has led to a conscious shift away from dependence on depleting crude oil feedstocks. Subsequently, production of biodiesel, a renewable and sustainable alternative fuel, has increased over the past years. Since 2008, biodiesel production has increased by 12% worldwide, reaching 716,000 barrels of oil equivalent per day in 2020 (BP 2021). Typically, production of biodiesel involves the transesterification of renewable vegetable oils or animal fats with methanol to yield fatty acid methyl esters (FAMEs) and the byproduct glycerol.^1^ With crude glycerol accounting for 10 wt % of biodiesel production, a glut in the glycerol market has occurred as a direct consequence of the increase in production.^2^ However, the cheap cost of glycerol (USP grade $0.9/kg) and its reactive nature make it a promising platform chemical.^3^ Consequently, increased attention has been placed on research and development of different pathways of glycerol value addition such as carbonation, dehydration, oxidation, and hydrogenolysis.^4^

Glycerol can also undergo esterification with carboxylic acids. The esterification of glycerol with acetic acid leads to the formation of monoacetin, diacetin, and triacetin glycerol esters as products. Industrial uses of these products include plasticizing agents and the production of biodegradable polyesters.^3^ The mixture of di- and triacetin can also be used as a fuel additive, improving the cold flow and viscosity properties of biodiesel and leading to a reduction in carbon monoxide, carbon dioxide, hydrocarbons, and nitrous oxides produced during combustion.^5,6^

While homogeneous mineral acid catalysts such as sulfuric and hydrochloric acid are highly active for the reaction, their nonreusable and corrosive nature can prove disadvantageous.^7^ As shown in previous work, ionic liquids can overcome these disadvantages; however, a multiple-step catalyst recovery could prove cumbersome on an industrial scale.^8^ To overcome some of the disadvantages of homogeneous catalysis, the use of heterogeneous catalysis has also been investigated. Solid acid catalysts such as metal oxides,^9−11^ ion-exchange resins,^12−15^ zeolites,^16−18^ and carbon-based catalysts^18−20^ have all been investigated for this reaction.

Heteropolyacids are another group of solid acid catalysts known for their strong Brønsted acidity, high activity and stability, and high water tolerance.^7^ Zhu et al. investigated glycerol esterification using three zirconia supported heteropolyacids: silicotungstic (STA), tungstophosphoric (TPA, DTP), and phosphomolybdic (PMA).^21^ Previously Zhu et al. reported that ZrO_2_ supported STA was the most active and had the highest stability when compared to supports such as y-Al_2_O_3_, activated carbon, TiO_2_, and SiO_2_.^22^ The protons of heteropolyacids can also be exchanged with metal ions such as K^+^, Cs^+^, Ag^+^, and Sn^2+^ to form heteropolyacid salts.^23,24^ While these salts are solid, some can remain slightly soluble in the reaction mixture, and it can be desirable to use a support for ease of separation. Tin exchanged heteropolyacids supported on K-10 montmorillonite clay have been shown to be effective catalysts for acid catalyzed reactions.^25^

To date, there has been limited investigation of the kinetics of the esterification of glycerol with acetic acid, with only a limited number of papers published on the topic. Most kinetic models developed for the reaction follow the Langmuir–Hinshelwood (LHHW) model.^26−29^ The studies have focused mainly on heteropolyacid and ion-exchange resin catalysts such as Purolite CT-275 and Amberlyst-15. However, Reinoso et al. found that the Eley–Rideal kinetic model suited their data utilizing the Dowex Monosphere 650 C best.^30^

In this work, tin exchanged tungstophosphoric acid supported on K-10 montmorillonite clay has been prepared and investigated for the esterification of glycerol with acetic acid. The effect of tin substitution on the catalyst activity and stability was investigated. The catalysts were characterized, and the effect of various process parameters was investigated with the view of maximizing the yield of the triacetin productivity. A kinetic model for the esterification of glycerol using tin exchanged tungstophosphoric acid supported on K-10 as the catalyst was developed and validated.

## 2 Experimental Section

### 2.1 Materials and Methods

All chemicals used were of analytical reagent grade and were used without further purification as commercially available. Acetic acid, montmorillonite K-10 clay, tungstophosphoric acid hydrate, and tin chloride were obtained from Sigma-Aldrich. Glycerol and methanol were obtained from Alfa-Aesar.

### 2.2 Catalyst Synthesis

The catalysts were prepared in a two-step incipient wetness impregnation method similar to that reported by Tiwari et al.^25^ In the first step, the required amount of tin(II) chloride (typically 0.027 g) was dissolved in methanol (2.5 mL) and added in small amounts (approximately 0.5 mL) to the required amount of K-10 clay (typically 1.6 g), the contents were mixed until dry, and then more solution was added. The solid catalyst was then dried in the oven at 120 °C for 4 h. In the second step, the calculated amount of tungstophosphoric acid (DTP) (typically 0.4 g) was dissolved in methanol (2.5 mL) and then loaded onto the solid catalyst in a similar procedure to that described above. The final catalyst was then dried at 120 °C for 4 h and then calcined at 300 °C for 4 h. Nonexchanged tungstophosphoric acid catalysts supported on K-10 were prepared by using the second step of the above procedure only, to give 20 wt % DTP/K-10 catalysts.

### 2.3 Catalyst Characterization

The prepared catalysts were characterized using X-ray Diffraction, Fourier-Transform Infrared Spectroscopy (FTIR), N_2_ sorption analysis, and acidity measurements. X-ray diffraction measurements were recorded using a Panalytical X-Pert Pro MPD diffractometer with Ni filtered CuKα radiation (1.5405 Å) with a step size of 0.016° from 5° to 80°. Fourier-transform infrared (FT-IR) spectra of neat catalyst samples were recorded using an Agilent Cary 630 FTIR spectrometer. Brønsted-acidity measurements were determined using an acid–base titration method. UV–visible spectra were collected using an Agilent Cary 60 UV–vis spectrophotometer, using a 0.33 mg/mL catalyst in deionized water. The catalysts were stirred in 25 mL of a 0.1 M NaOH solution for 6 h and then titrated with a 0.1 M HCl solution to calculate the catalyst Brønsted-acidity. The surface area, total pore volume, and average pore diameter were measured by N_2_ adsorption–desorption isotherms at 77 K using a Micromeritics ASAP 2020. The pore size was calculated on the adsorption branch of the isotherms using the Barrett–Joyner–Helenda (BJH) method, and the surface area was calculated using the Brunauer–Emmett–Teller (BET) method.

### 2.4 Catalyst Activity Testing

All reactions were performed in a 100 mL glass reactor equipped with baffles, a magnetic stirrer, and a condenser. Typically, glycerol (5 g, 0.054 mol), acetic acid (32.42 g, 0.54 mol), and a catalyst (10 wt % with respect to glycerol) were loaded into the reactor. The reactor was placed into an isothermal oil bath at a known temperature (typically 110 °C) and agitated for the required period. Samples were withdrawn periodically for analysis. An Agilent 7820A GC equipped with a HP-5 capillary column and FID detector were used to analyze the reaction samples.

## 3 Results and Discussion

### 3.1 Catalyst Characterization

**Figure 1:**
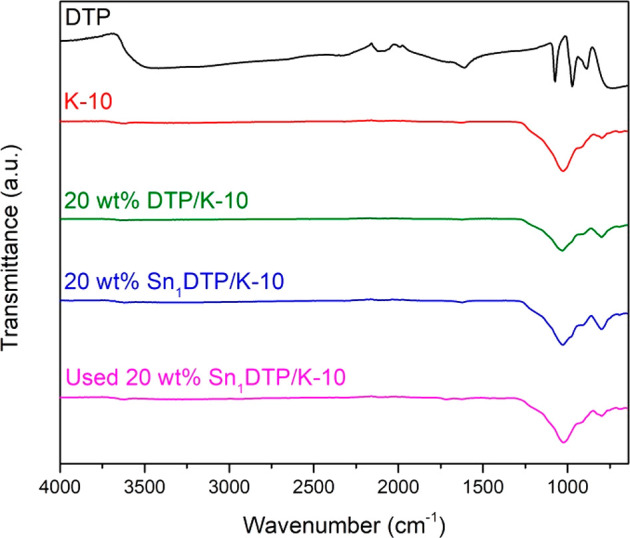
FT-IR spectra of prepared catalysts.

The FT-IR spectra of the catalysts are shown in Figure 1. The Keggin structure of DTP is clearly shown through the characteristic bands present at 768, 891, 966, and 1068 cm^–1^.^31^ The FT-IR spectra of the supported catalysts show similar peaks to that of pristine K-10 clay, with a distinct band and shoulder at 1030 and 913 cm^–1^, respectively. The similar nature of the spectra can be attributed to the overlap of the distinctive K-10 bands with the bands associated with the Keggin structure of DTP.^32^ The spectra of the four times used 20 wt % Sn_1_DTP/K-10 catalyst are also present, which shows no discernible difference than the fresh catalyst, indicating the Keggin structure remains stable under the reaction conditions.

**Figure 2:**
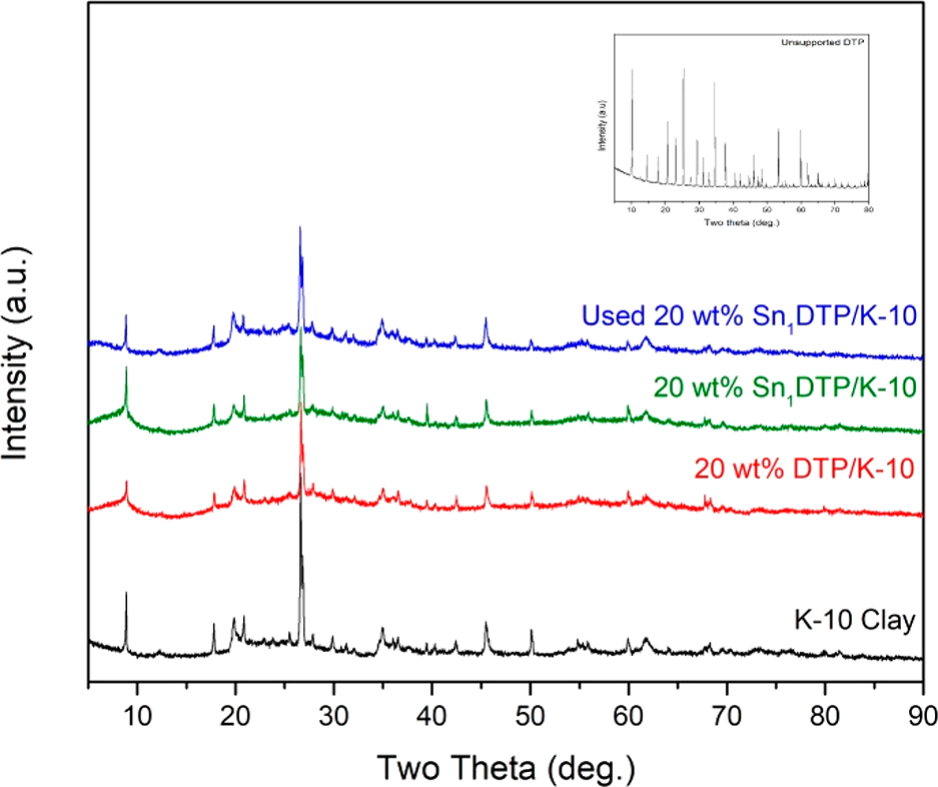
X-ray diffraction patterns of the prepared catalysts: (a) K-10 clay, (b) 20 wt % DTP/K-10, (c) fresh 20 wt % Sn_1_DTP/K-10, and (d) used 20 wt % Sn_1_DTP/K-10. (e) The inset showing the characteristic XRD pattern of unsupported DTP.

The X-ray diffraction patterns of the prepared catalysts are shown in Figure 2. Pristine K-10 is highly crystalline in nature and shows peaks related to montmorillonite and other impurities such as quartz, feldspar, and phengite.^25^ Like the FT-IR spectra, the XRD spectra of DTP and Sn_1_DTP supported on K-10 clay are similar in nature to that of pristine K-10, due to a uniform distribution of the active component on the surface of the clay. Furthermore, the XRD pattern of the 4 times used catalyst was highly similar to that of the fresh 20 wt % Sn_1_DTP/K-10 catalyst, indicating the stability of the catalyst under the reaction conditions.

**Figure 3:**
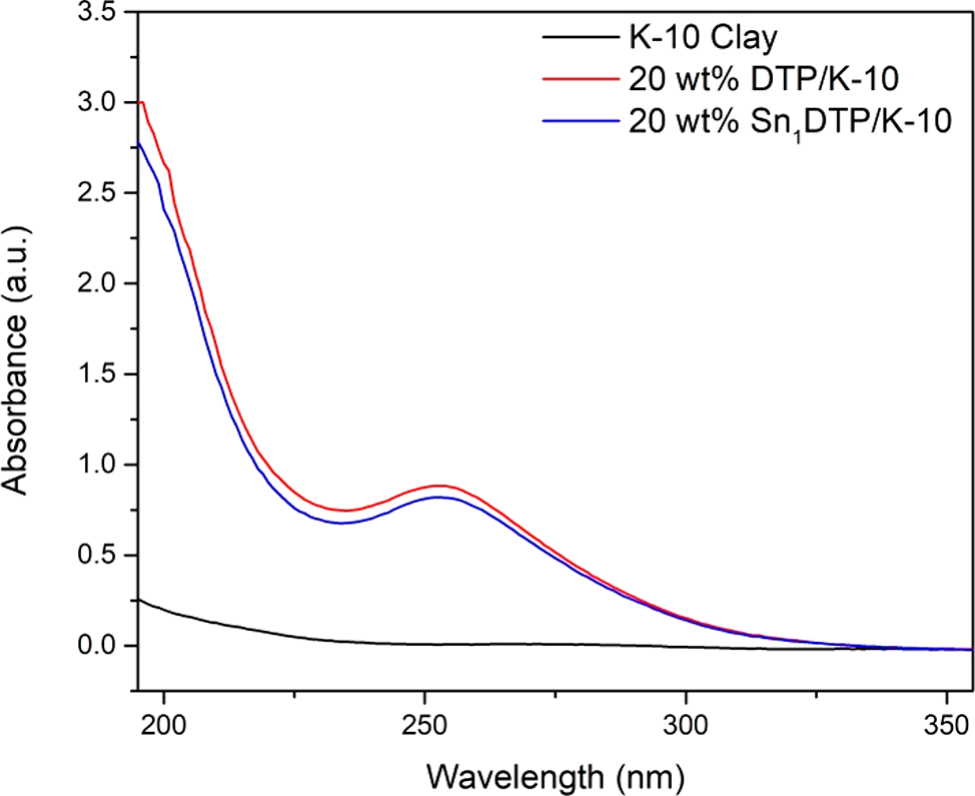
UV–visible spectra of (a) K-10 clay, (b) 20 wt % DTP/K-10, and (c) 20 wt % Sn_1_DTP/K-10.

UV–visible spectra of the prepared catalysts are shown in Figure 3. The K-10 spectra show no absorption bands, which indicate that no absorbance of light is occurring. Both DTP/K-10 and Sn_1_DTP/K-10 show two distinct absorption bands at 190 and 253 nm, corresponding to charge transfer from the terminal oxygen and bridge oxygen to the metallic tungsten center, respectively.^32^ The presence of these peaks in Sn_1_DTP/K-10 indicates that the Keggin ion structure remains intact upon exchanging the protons of DTP with tin.

The BET surface area, pore diameter, and pore volume for different samples are listed in Table 1. K-10 has a high surface area which decreases after the loading of DTP. The pore volume of the supports also decreased due to the filling of pores of K-10 with the DTP. The surface area of the Sn_*x*_-DTP/K-10 catalyst increases with exchange of Sn. The formation of the dense porous network between Sn and DTP could result in an increase in the surface area. The pore size of the catalyst was found to be in the range of 5.1–7.2 nm, which indicates that the catalysts are mesoporous in nature.

**Table 1:** Surface Area Pore Volume and Pore Diameter Analysis

no.	catalyst	BET surface area (m^2^/g)	pore volume (cm^3^/g)	pore diameter (nm)
1	K-10 clay	227.2	0.38	5.85
2	20% (w/w) DTP/K-10	106.9	0.28	7.52
3	20% (w/w) Sn_0.5_DTP/K-10	146.4	0.23	7.48
4	20% (w/w) Sn_1_DTP/K-10	155.1	0.23	7.23
5	20% (w/w) Sn_1.5_DTP/K-10	161.1	0.22	7.21

### 3.2 Initial Catalyst Screening

**Figure 4:**
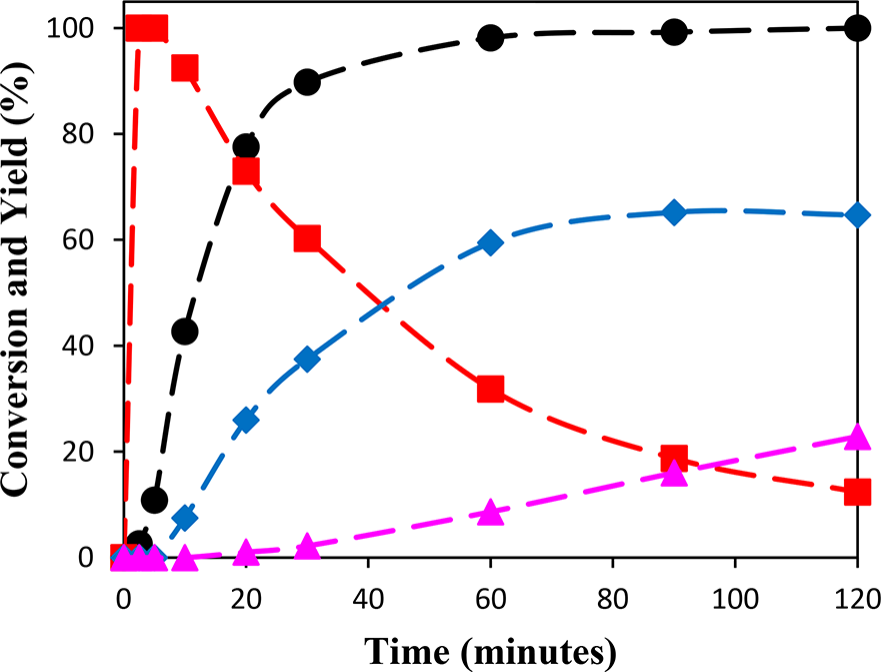
Reaction-time profile for glycerol esterification with acetic acid using Sn_1_DTP/K-10 as the catalyst; (●) % conversion of glycerol, (■ (red)) % yield of monoacetin, (◆ (blue)) % yield of diacetin, and (▲ (purple)) % yield of triacetin. Reaction conditions: glycerol 0.054 mol, acetic acid 0.54 mol, 10 wt % catalyst, 110 °C, 800 rpm.

The activity of the prepared catalysts was screened in a batch reaction of glycerol and acetic acid, with the results shown in Table 2. All catalysts gave complete conversion of glycerol in 2 h, except for K-10 clay which gave an ∼97% conversion. As a result, product yield was chosen as an effective way to compare the activity levels of the catalysts. A typical reaction-time profile is shown in Figure 4, where the stepwise conversion of monoacetin to diacetin and diacetin to triacetin can be seen.

**Table 2:** Effect of Various Catalysts on the Esterification of Glycerol with Acetic Acid^a^

		product yield (%)			
catalyst	glycerol conversion (%)	monoacetin	diacetin	triacetin	acidity^c^(mmol g^–1^)
K-10	96.7	44.1	47.5	5.1	0.82
DTP/K-10	100.0	6.6	67.9	25.5	1.45
Sn_0.5_DTP/K-10	100.0	7.8	69.1	23.2	1.85
Sn_1.0_DTP/K-10	100.0	6.9	64.9	28.2	2.21
Sn_1.5_DTP/K-10	100.0	10.1	64.5	25.4	1.25
DTP^b^	100.0	2.4	45.7	51.9	-

a Reaction conditions: glycerol (0.054 mol), acetic acid (0.54 mol), 10 wt % catalyst loading, 800 rpm, 110 °C, 120 min.

b DTP loading was 0.1 g.

c Acidity was measured using titration with a 0.1 M sodium hydroxide solution.

Pristine K-10 provided the lowest level of activity, due to its low acidity levels (0.82 mmol g^–1^), with only a 5.1% yield of triacetin. Supporting 20 wt % DTP on the surface of K-10 results in an increase in acidity to 1.45 mmol g^–1^, with the yield of triacetin increasing to 25.5%. The effect of DTP proton exchange with Sn on the yield of triacetin was examined. Exchange of a proton with Sn (*x* = 0.5) resulted in an increase in the acidity of the catalyst to 1.85 mmol g^–1^. However, this increase did not result in an increase in the triacetin yield from unexchanged DTP/K-10 with a 23.2% yield of triacetin observed. Replacing another H^+^ with Sn (*x* = 1.0) results in an increase in acidity to 2.21 mmol g^–1^, the highest acidity of all the prepared catalysts. The high increase in acidity resulted in an increase in a triacetin yield of 2.7% from unexchanged DTP/K-10 to a 28.2% triacetin yield. Complete proton exchange with Sn (*x* = 1.5) resulted in a decrease in acidity to 1.25 mmol g^–1^. In partially exchanged heteropolyacids, the mobility of residual protons is known to be higher and exhibits increased Bronsted acidity. Also, exchanging the protons of the coordinately unsaturated Sn^2+^ species generates Lewis acidity. The ratio of Bronsted to Lewis acidity varies depending on the extent of Sn exchanged. Thus, partially exchanged heteropolyacid catalysts show higher overall acidity compared to fully exchanged catalyst. The triacetin yield of 25.4% is close to the unexchanged DTP/K-10 catalyst; however, a lower yield of diacetin is obtained with 64.5% compared to 67.9%, respectively, and an increase in the monoacetin yield is obtained with 10.1% compared with 6.6%, respectively. As a result of the initial screening activity, Sn_1.0_DTP/K-10 was chosen as the catalyst for further investigation due to the high level of acidity and highest selectivity to triacetin.

### 3.3 Effect of Reaction Parameters

#### 3.3.1 Effect of Stirrer Speed

**Figure 5:**
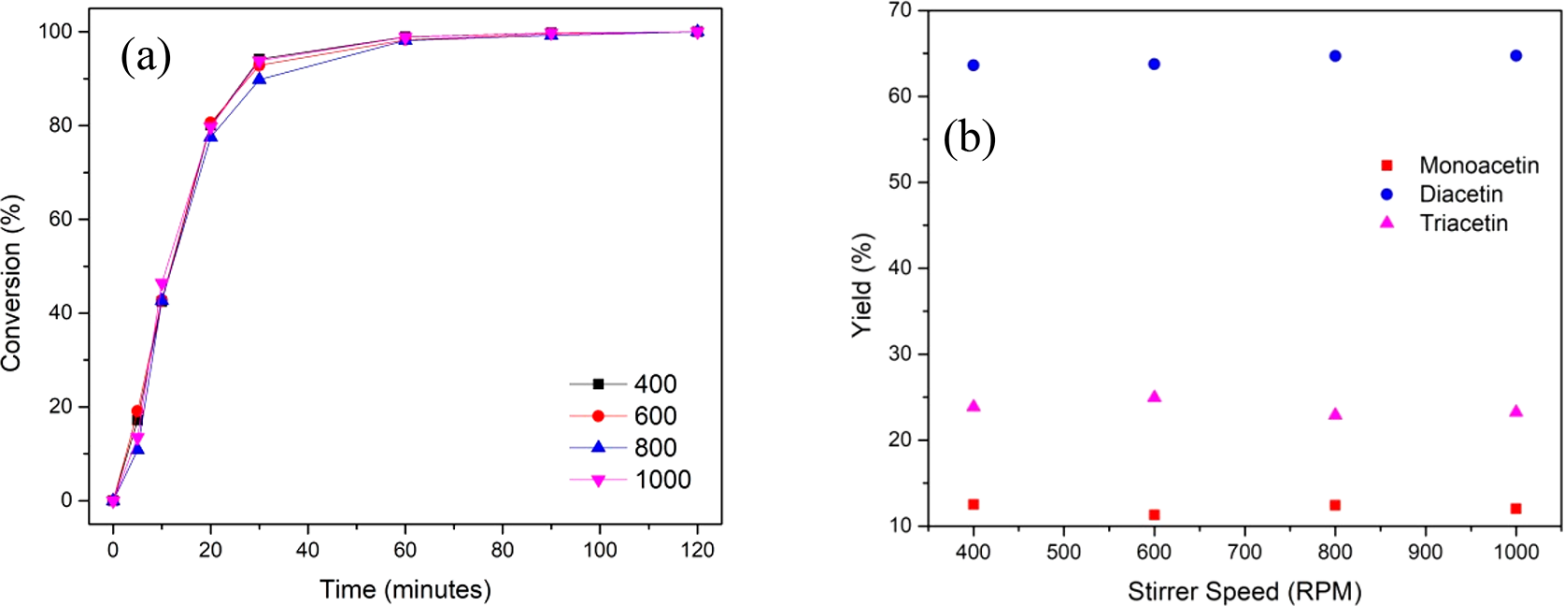
Effect of stirrer speed on (a) glycerol conversion and (b) product yield. Reaction conditions: glycerol (0.054 mol), acetic acid (0.54 mol), 10 wt % catalyst, 110 °C, 120 min.

The effect of stirrer speed was investigated in the range of 400 to 1000 rpm, with the results shown in Figure 5. It was found that the agitation rates had no effect on either the rate of glycerol conversion or the product distribution. From this, it was confirmed that all experiments were performed with no external mass transport limitations present, with the rate at which the reactant species transfers from the bulk liquid phase to the external surface of the catalyst faster than that of the observed reaction rate.

#### 3.3.2 Effect of Catalyst Loading

**Figure 6:**
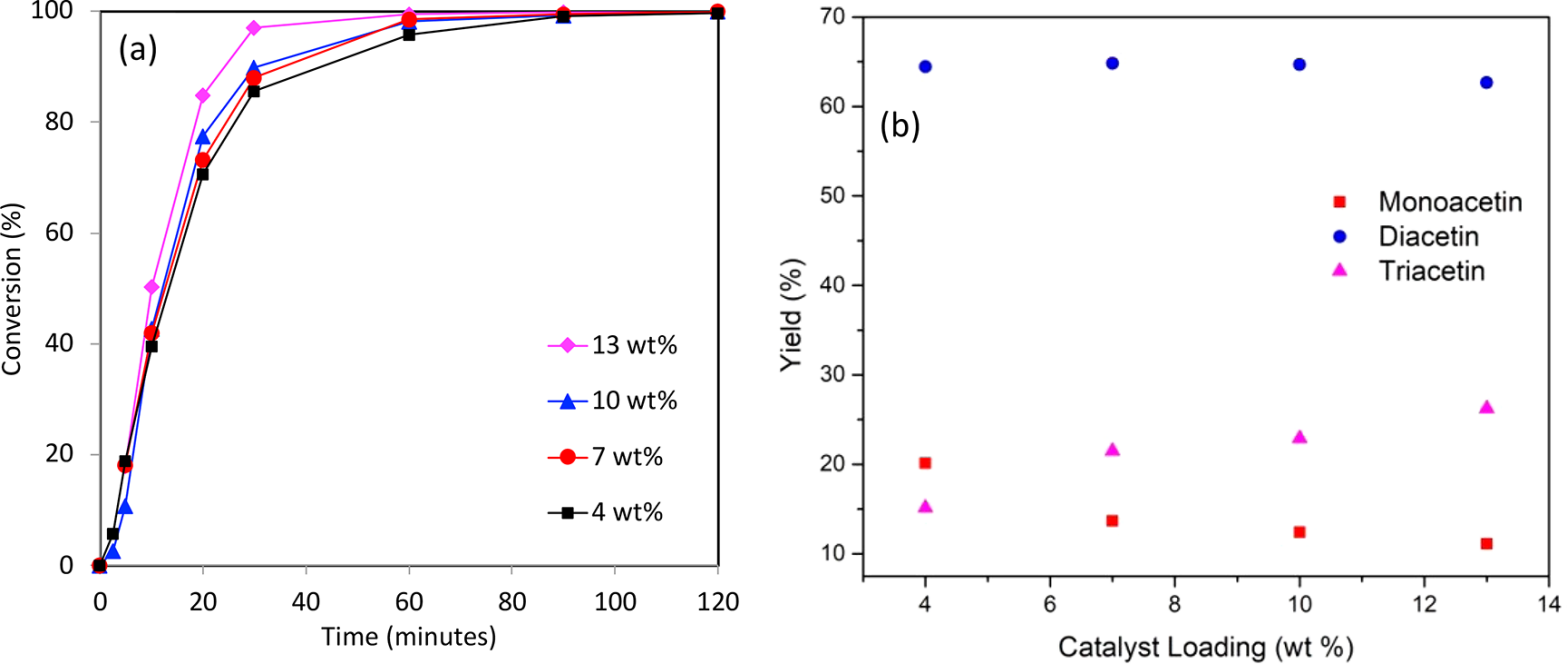
Effect of catalyst loading on (a) conversion and (b) product yield using a Sn_1.0_DTP/K-10 catalyst. Reaction conditions: glycerol (0.054 mol), acetic acid (0.54 mol), 110 °C, 800 rpm, 120 min.

The effect of catalyst loading was examined at 4 different levels: 4, 7, 10, and 13 wt % catalysts (Sn_1.0_DTP/K-10) with respect to glycerol being the limiting reactant. As can be seen in Figure 6, increased catalyst loading leads to an increase in the yield of triacetin. This is attributed to the increase in the number of available active sites of the catalyst, with a corresponding increase in the catalyst loading. Triacetin yields an increase from 15.2% using a 4 wt % catalyst to 26.2% using a 13 wt % catalyst. While all catalyst loadings gave 100% glycerol after 120 min, increased catalyst loading also increased the rate of glycerol consumption. Glycerol conversion increased after 20 min by 14.1% to 84.8% when increasing the loading from a 4 wt % to a 13 wt % catalyst.

#### 3.3.3 Effect of Temperature

**Figure 7:**
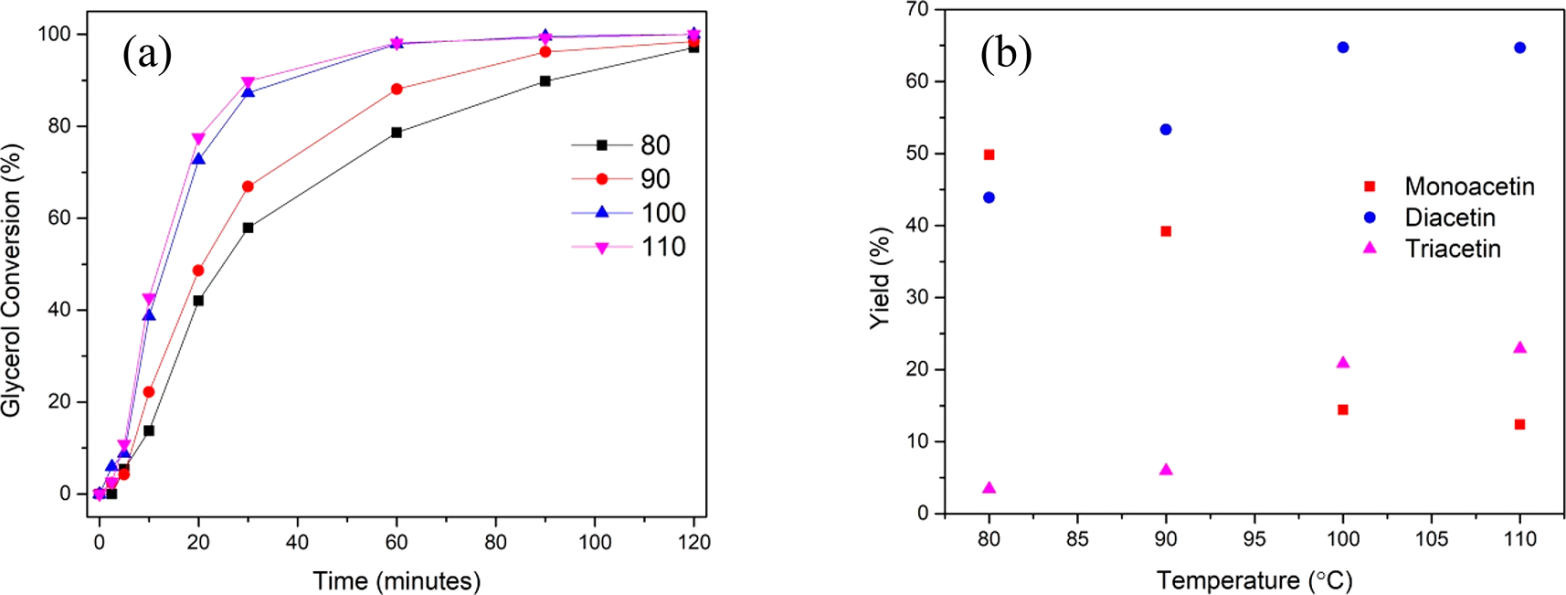
(a) % Conversion of glycerol and (b) product yields (%) at various reaction temperatures. Reaction conditions: glycerol (0.054 mol), acetic acid (0.54 mol), 10 wt % catalyst loading, 800 rpm, 120 min.

The effect of temperature was investigated in the range of 80 to 110 °C. The top temperature range was chosen to be 110 °C, with Mufrodi et al. identifying that above 115 °C uncontrolled acetic acid evaporation leads to a decrease in triacetin yield.^33^ The effect of temperature on glycerol conversion and product yield is shown in Figure 7.

It can be seen that temperature has both a profound effect on the rate of glycerol conversion and product distribution. Increasing the temperature from 90 °C to 100 °C had the biggest effect out of the temperature range tested. After 20 min, glycerol conversion is 48.6% at 90 °C which is increased to 72.7% at 100 °C. Similarly, for the triacetin yield, at 90 °C, the yield is low at 6.0% but increases dramatically to 20.8% at 100 °C. When increasing the temperature from 100 °C to 110 °C, no significant change in glycerol conversion or product distribution was observed. The increase in the conversion and the yield with increasing temperature also indicates that under given reaction conditions, there are no mass transfer limitations.

#### 3.3.4 Effect of Glycerol on the Acetic Acid Mole Ratio

**Figure 8:**
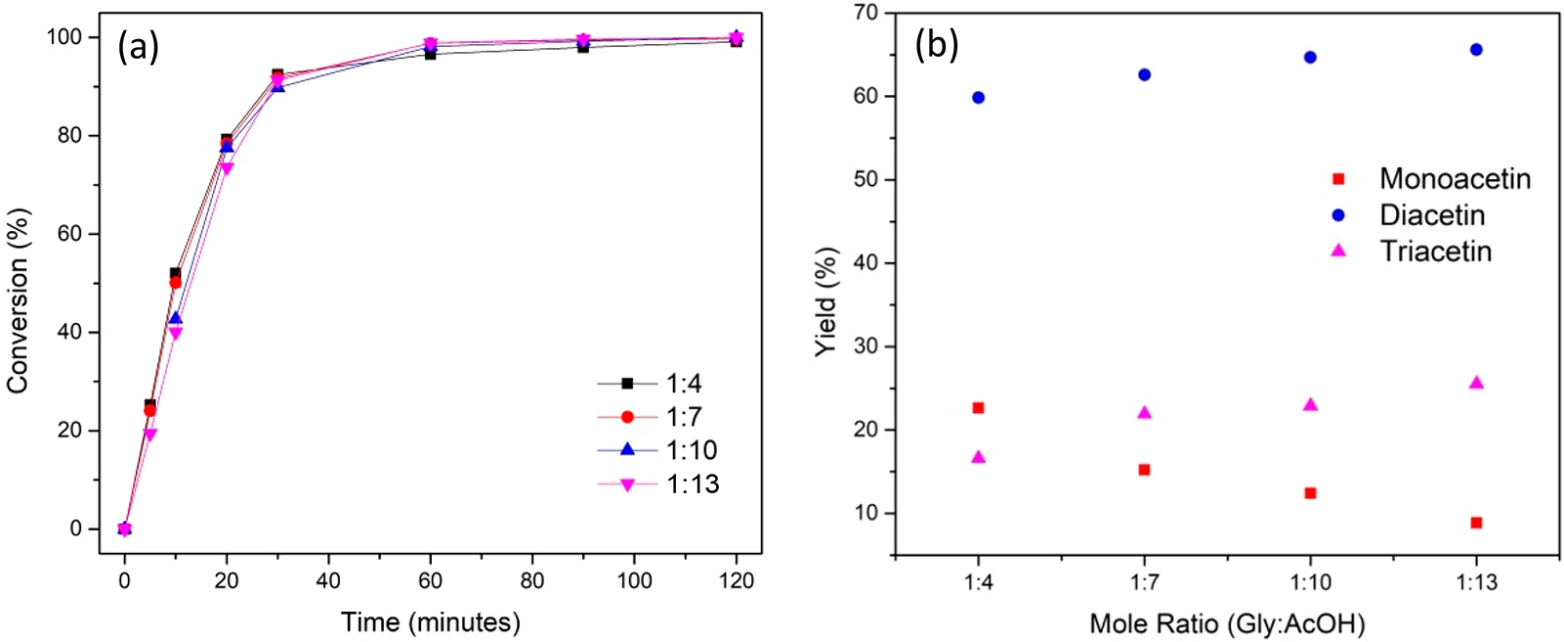
Effect of the mole ratio on (a) glycerol conversion and (b) product yield. Reaction conditions: glycerol (0.054 mol), 10 wt % catalyst loading, 110 °C, 800 rpm, 120 min.

The effect of glycerol on the acetic acid mole ratio was examined in a range from 1:4 to 1:13. The effect of the mole ratio on the product yield is shown in Figure 8. Interestingly, the mole ratio had very little effect on the rate of glycerol conversion, with temperature and catalyst loading having a greater effect on this. However, the effect of the mole ratio on the product yield is apparent, with a greater mole ratio leading to a substantial decrease in the amount of monoacetin present in the mixture and an increase in the yield toward triacetin. Increasing the mole ratio from 1:4 to 1:13 increases the yield of triacetin from 16.6% to 25.5%. As the formation of 1 mol of triacetin requires 3 mol of acetic acid, hence with an increase in the mole ratio, the amount of acetic acid with respect to glycerol increases, and hence the yield of the formation of diacetin and triacetin also increases.

### 3.4 Catalyst Reusability

To assess the reusability of the Sn_1.0_DTP/K-10 catalyst in the esterification reaction, the used catalyst was separated from the reaction mixture and subsequently dried overnight in an oven at 100 °C. The dried catalyst was then used again in a further reaction to assess the stability.

**Figure 9:**
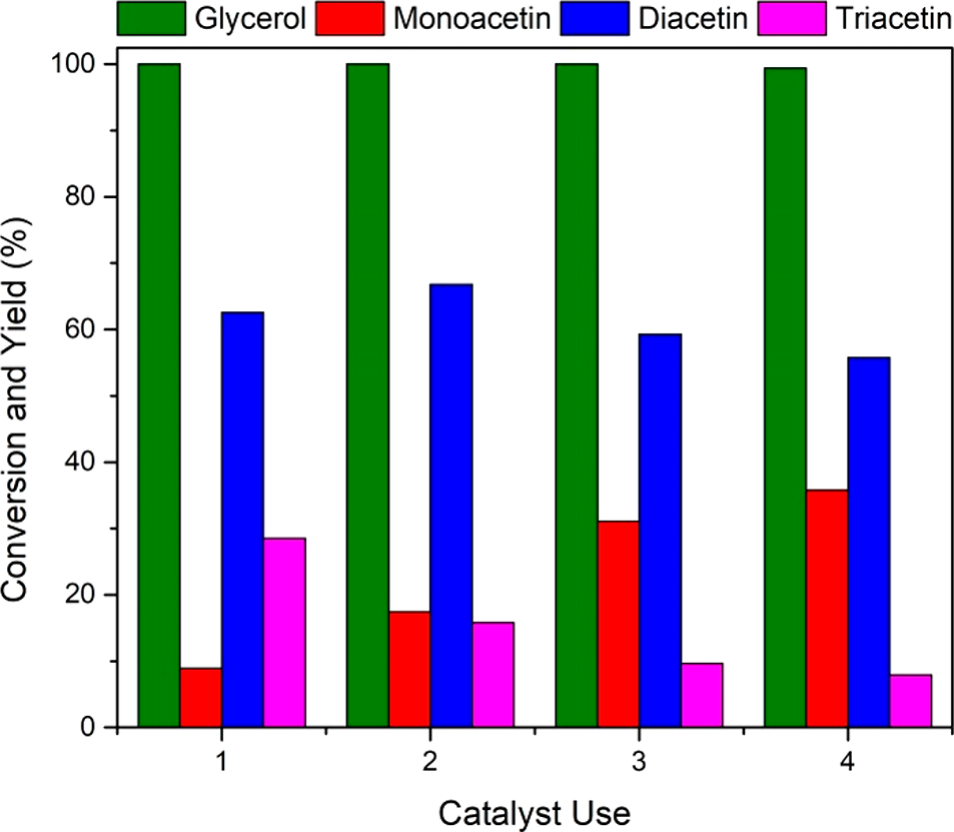
Effect of Sn_1.0_DTP/K-10 reusability on glycerol conversion and product yield. Reaction conditions: glycerol (0.054 mol), acetic acid (0.54 mol), 10 wt % catalyst, 110 °C, 800 rpm, 120 min.

As the results in Figure 9 show, Sn_1.0_DTP/K-10 shows good recyclability. Glycerol conversion remains constant over each of the 4 catalytic cycles. Some deviation in product distribution occurs with decreasing the yield of triacetin on each recycle. Similarly, the monoacetin yield increases upon each reuse of the catalyst.

### 3.5 Development of Kinetic Model

The above results were used to build a kinetic model of the reaction, with a Langmuir–Hinshelwood (L-H) dual-site mechanism proposed and derived. The effect of internal diffusion on the process kinetics was evaluated using the classical Weisz–Prater criterion (*C*_*WP*_), which represents the ratio of the intrinsic reaction rate to the intraparticle diffusion rate, and can be evaluated from the observed rate of reaction, the particle radius *R_p_*, effective diffusivity of the limiting reactant *D_e_*, and concentration of the reactant at the external surface of the particle. The Wiesz–Prater criterion, the dimensionless parameter *C_WP_*, can be calculated as below
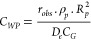
1where *r*_*obs*_ = observed reaction rate (mol kg^–1^ s^–1^), ρ_*p*_ = catalyst density (kg m^–3^), *R*_*p*_ = catalyst particle radius (m), *D*_*e*_ = effective diffusivity (m^2^ s^–1^), and *C*_*G*_ = bulk liquid glycerol concentration (mol m^–3^).

The effective diffusivity (*D*_*e*_) was determined with the following equation:
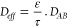
2

Conservative estimates for porosity and tortuosity of the catalyst were taken as 0.23 and 3, respectively. The Weisz–Prater criterion was calculated at different temperatures, as shown in Table 3. In the present case, the values of *C*_*WP*_ were much less than 1, therefore, indicating that there was no intraparticle diffusion resistance and the reaction is intrinsically kinetically controlled. For a L-H dual site model, the global reaction order is 2. As such, Mears postulated conservative limits for such reactions, stipulating a value of lower than 0.3 required to confirm the absence of internal diffusion limitations. The criterion is well satisfied, indicating the lack of such limitations.^31^

**Table 3:** Calculated Weisz–Prater Criterion at Different Reaction Temperatures

reaction temp (°C)	*r*_*obs*_(mol cm^-3^ s^-1^)	*D*_*AB*_(cm^2^/s)	*C*_*WP*_
80	0.638 × 10^–6^	4.15 × 10^–06^	6.0 × 10^–5^
90	0.806 × 10^–6^	4.73 × 10^–06^	6.73 × 10^–5^
100	1.345 × 10^–6^	5.36 × 10^–06^	9.96 × 10^–5^
110	1.457 × 10^–6^	6.05 × 10^–06^	9.73 × 10^–5^

It was assumed that there was weak adsorption of the reactant and product species, meaning that the resistance term can be omitted from the model. The surface reaction of the adsorbed species the catalyst was assumed to be the rate-determining step. The surface reactions were also assumed to be irreversible, due to the excess of acetic acid used in comparison with glycerol. The esterification of glycerol with acetic acid consists of three stepwise reactions to produce triacetin, as described below
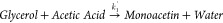
3
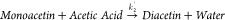
4
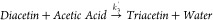
5where *k*′ is the apparent rate constant for the surface reaction between adsorbed species. With
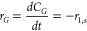
6
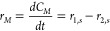
7
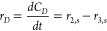
8
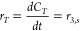
9where *G* = glycerol, *M* = monoacetin, *D* = diacetin, *T* = triacetin, *r* = rate of change of species, and C = concentration of species within the reactor.
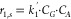
10
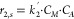
11
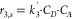
12

As glycerol is the only source of product formation within the reactor, the concentration balance at any time can be defined as follows:
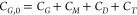
13

**Figure 10:**
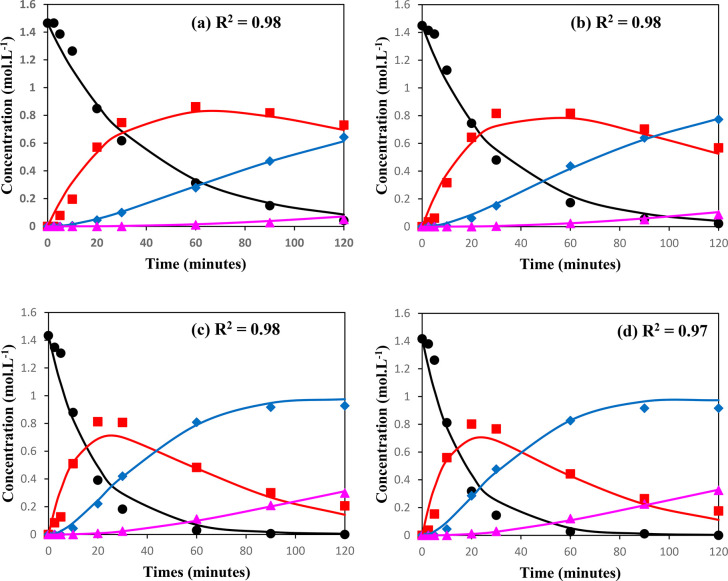
Reaction-time profiles at various temperatures. The points correspond to experimental values, and the solid lines are calculated values. (●, -) % conversion of glycerol, (■, - (red)) % yield of monoacetin, (◆, - (blue)) % yield of diacetin, and (▲, - (purple)) % yield of triacetin. Reaction conditions: glycerol (0.054 mol), acetic acid (0.54 mol), 10 wt % catalyst loading. Temperature: (a) 80 °C, (b) 90 °C, (c) 100 °C, (d) 110 °C.

To compute the apparent rate constant (*k*′), a Matlab nonlinear least-squares method was employed, utilizing the function *Isqcurvefit*. The computed rate constants are shown in Table 4, and predicted model concentrations and experimental concentrations at various temperatures are shown in Figure 10.

**Table 4:** Computed Rate Constants

	temperature (°C)			
rate constants (L mol^–1^ min^–1^)	80	90	100	110
*k*_1_^′^	1.79 × 10^–3^	2.32 × 10^–3^	3.95 × 10^–3^	4.38 × 10^–3^
*k*_2_^′^	6.36 × 10^–4^	8.97 × 10^–4^	2.04 × 10^–3^	2.24 × 10^–3^
*k*_3_^′^	1.61 × 10^–4^	1.76 × 10^–4^	3.33 × 10^–4^	3.45 × 10^–4^

**Figure 11:**
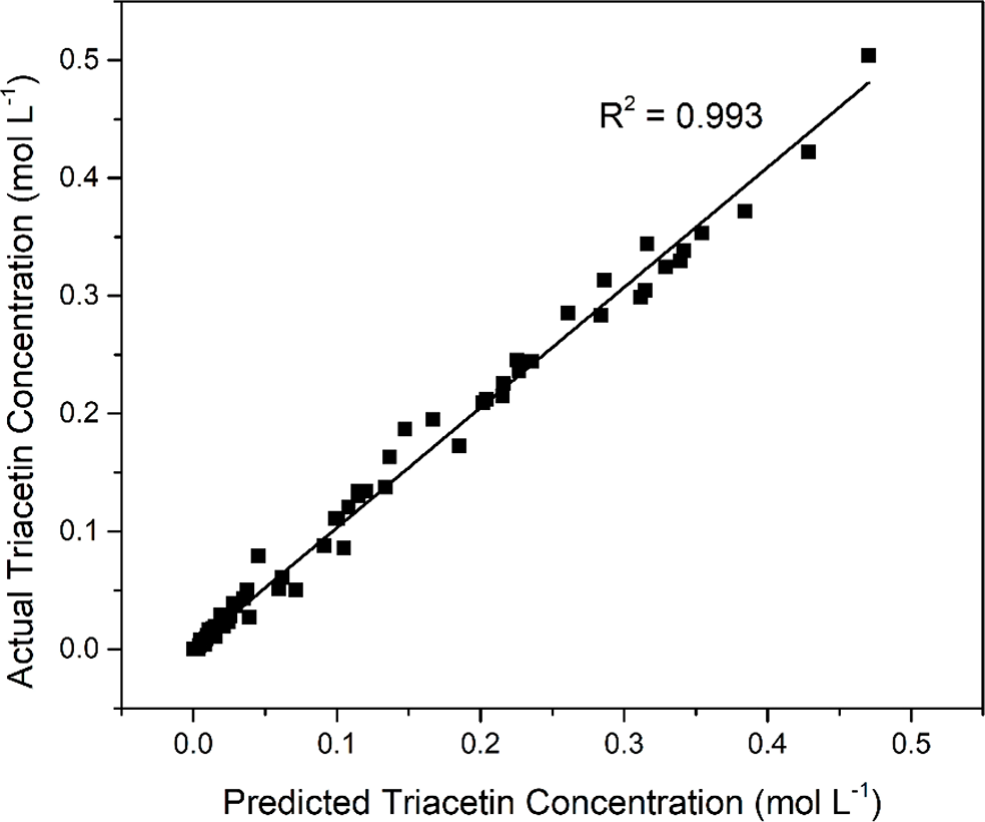
Parity plot showing experimental vs predicted triacetin concentration values.

Good agreement was found between the predicted model concentrations and the experimental concentrations. This is further quantified through the calculation of global *R*^2^ values, which over the range of temperatures tested was a minimum of 0.97. This accuracy of the kinetic model can be further observed with the parity plot of predicted and experimental triacetin concentration shown in Figure 11. The parity plot gives an *R*^2^ value of 0.993 indicating high agreement.

Through completing the model fitting procedure utilizing experimental data conducted at various temperatures, Arrhenius plots can subsequently be constructed, enabling the graphical determination of the activation energies for each of the esterification reactions which are shown in Table 5. As can be seen in Table 5, the activation energy required increases at each esterification step, indicating an increased sensitivity to temperature. This is reflected in the experimental results where increasing the reaction temperature from 90 to 100 °C had a profound impact on the selectivity to triacetin.

**Table 5:** Esterification Activation Energies

esterification step	activation energy (kJ mol^–1^)
1	53
2	65
3	94

The activation energies reported are higher than those reported by Veluturla et al. using a cesium exchanged tungtstophosophoric acid catalyst.^26^ Under a 9:1 acetic acid to glycerol ratio, with a 5 wt % catalyst at 110 °C the activation energy for each step was found to be 24.99, 28.10, and 51.73 kJ mol^–1^, respectively. Similar values for the first step of the reaction were reported by Patel et al. using tungstophosphoric acid supported on MCM-41 and zirconia.^34^ With an acetic acid to glycerol ratio of 1:6 and 0.15 g catalyst loading at 100 °C, the activation energy was found to be 22.3 and 25.2 kJ mol^–1^ for each catalyst, respectively. Similar high activation energies have been reported using tungstophosphoric acid anchored to MCM-48, with the first step activation energy of 46.8 kJ mol^–1^ reported.^35^

## 4 Conclusions

Catalysts of DTP and tin exchanged DTP supported on K-10 were prepared. It was found that Sn_1_DTP/K-10 was the most active catalyst for the reaction of glycerol with acetic acid, obtaining high levels of glycerol conversion and a high yield of triacetin. The effect of the reaction parameters was studied, and it was found that temperature and the mole ratio of glycerol to acetic acid had the most profound effect on the yield of triacetin. A high catalyst loading was also required in order to facilitate high yields of triacetin. The catalyst shows good recyclability; however, a decrease in the triacetin yield is observed over the four reaction cycles. A kinetic model of the reaction was fitted, and the Langmuir–Hinshelwood (L-H) dual-site model was able to describe the experimental data with high agreement between the experimental and calculated results.
